# *Toxocara cati* larva migrans in domestic pigs - detected at slaughterhouse control in Norway

**DOI:** 10.1186/1751-0147-54-66

**Published:** 2012-11-21

**Authors:** Rebecca K Davidson, Anna Mermer, Øivind Øines

**Affiliations:** 1 Norwegian Veterinary Institute, Pb 750 Sentrum, 0106, Oslo, Norway; 2 Nortura Rudshøgda AS, Postboks 70, 2360, Rudshøgda, Norway

**Keywords:** Fattening pigs, Internal transcribed spacer, Larva migrans, Meat inspection, *Toxocara cati*, Zoonosis, 5S ribosomal DNA

## Abstract

Routine *Trichinella* meat inspection at the slaughterhouse detected one larva in a pooled batch of 100 pig samples. The larva was sent to the Norwegian Veterinary Institute (NVI) for species identification.

Morphological examination revealed that the larva was not *Trichinella* spp. Molecular analysis was performed. PCR and sequencing of 5S/ITS identified the larva as *Toxocara cati*. A second round of digests was carried out at the meat inspection laboratory, in smaller batches to try to identify the infected animal. No further larvae were detected and it was not possible to identify which of the 100 animals the larva had come from. This is the first time that *Toxocara cati* has been reported in slaughterhouse pigs in Norway.

Although the infected individual could not be identified, the meat originated from one of six potential farms. A small survey regarding rodent control and cats was sent to each of these farms. Cats had restricted access to food storage areas (two farms reported that cats had access) whilst none of the farms allowed cats into the production housing. Cats were, however, present on all the farms (mostly stray cats of unknown health status). Half of the farms also reported seeing rodents in the pig housing during the previous six months and half reported finding rodents in the feed and straw storage areas. We were unable to narrow down the source of infection – however contamination of food or bedding material, with cat faeces or infected rodents, in addition to the presence of infected rodents in pig housing remain potential routes of infection.

## 

Routine meat inspection is carried out on all pork intended for human consumption in Norway at meat inspection laboratories authorised by the Norwegian Food Safety Authority. *Trichinella* meat inspection is carried out in accordance with EU legislation [1]. A positive *Trichinella* finding in a pooled batch means that all the carcasses in that batch have to be withheld from further production until the infected individual can be identified. Suspected positive findings have to be sent to the National Reference Laboratory for *Trichinella*, in this case the Norwegian Veterinary Institute (NVI), for confirmation. Smaller pools of meat, from the initial batch of 100 individuals, must subsequently be examined to further narrow down the search for the infected individual prior to individual digests to identify the infected animal.

Theoretically the common differential diagnoses for larvae found during *Trichinella* digestion include *Trichinella* spp., *Ascaris suum* and *Metastrongylus apri*. Experimental infections with *Toxocara canis* have shown that pigs are suitable hosts and that larva migrans can occur with the liver and lungs being favoured sites for the larvae during early infection stages [2,3]. To the best of our knowledge there are no published cases of naturally infected pigs with *Toxocara cati* larva migrans, although pigs are listed as a suitable paratenic host [4] and *T. cati* eggs have been detected in the faeces of fattening pigs [5]. *Toxocara cati* larva migrans has been described in poultry [6] and *Toxocara* larvae in undercooked/raw meat dishes have been implicated in larva migrans in humans [7].

*Trichinella* was last suspected in Norwegian pigs in 1994 [8] – although the diagnosis was not confirmed by the National Reference Laboratory and was solely based upon the detection of larvae in the digest. The slaughterhouse was not able to trace which of the animals in the pooled digest were infected.

In spring 2012, a single larva was detected in a 100 sample pooled batch of pork at *Trichinella* meat inspection at the laboratory attached to the Nortura Rudshøgda slaughterhouse. Nortura contacted the National Reference Laboratory (NVI) and sent the larva for identification before initiating further investigation of the 100 animals in the initial batch. A second sample was taken by the authorised meat inspectors from the intercostal muscles of each of the animals from the initial batch. A second digest was carried out consisting of twenty digests each containing five 20 gram muscle samples. No larvae were detected during this second round of investigation. The carcasses were withheld from further production and were only released once the larva had been identified.

Limited morphological analysis of the larva was carried out using a stereomicroscope that unfortunately didn’t have measurement capability. Priority was given to isolating the larva for molecular analysis. The larva was motile upon examination at NVI. It was noticeably smaller than *Trichinella* larvae and had a brownish hue throughout the cuticle and internally. It did not have the distinctive anterior morphology typical of *Trichinella* larvae, namely discoid stichocytes occupying the anterior half of the body cavity [9]. We concluded that the larva was not *Trichinella*.

Molecular analysis was carried out to identify the larva to species level using both *Trichinella* PCR primers and universal primers. The primary aim of the molecular analysis was to corroborate the morphological diagnosis and the secondary aim was to identify the larva to species level. A PCR, using L6625/H7005 *Trichinella* primers [10] was set up, but failed to produce any products. A second PCR, using primers from the 5S intergenic spacer region [11], successfully generated a product. A third PCR was set up using the generic primers ITS-1 (5^′^-TTT CCG TAG GTG AAC CT-3^′^) and ITS-2 (5^′^-TCC TCC GCT TAG TGA TA-3^′^) and this also produced a product. The PCR products were visualised on a GelRed stained agarose gel (1.5%), and then cleaned using Nucleospin Gel and PCR cleanup (Mackerey-Nagel, Düren Germany). Sequencing was performed using BigDye 3.1 terminator mix and products were run on an ABI AVANT automated sequencer. Forward and reverse chromatograms were imported into Vector NTi (Invitrogen) and manually edited prior to assembly. The assembled molecules from the second and third PCR were included in a Blast search.

No identical matches in Genbank were found for the 479 bp product (JX014376) from the 5S PCR of the sample, but a sequence from *Toxocara canis* appeared at the top of the match list after performing a Blast search ( http://blast.ncbi.nlm.nih.gov/). The top hits were from the following organisms: *Toxocara canis* (TCU65503), *Ascaris lumbricoides* (M27961.1), *Chandlerella quiscali* HM641830), *Foleyella furcata (*AJ250988), *Mansonella ozzardi* (JF412308), *Onchocercidae* cf. *Mansonella ozzardi* (JF412312-13, JF412315-16). A maximum likelihood tree (ML) was constructed in MEGA [12] on an alignment of these molecules (Figure 1). No Genbank entries from 5S IGS for *Toxocara cati* were available. An alignment with the closest Genbank match TCU65503 (*T*. *canis*) revealed that this molecule was only 79.8% similar to the sequence from our sample, including several gaps in the sequence alignment.

**Figure 1:**
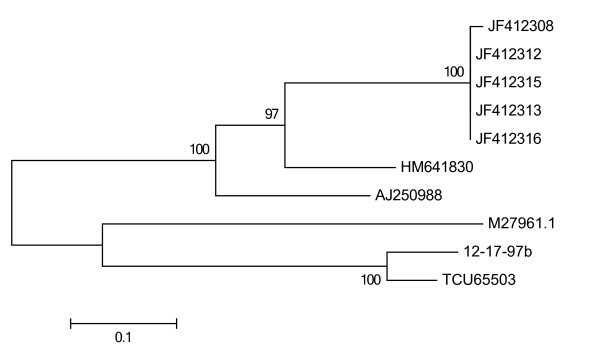
A maximum likelihood (ML) phylogenetic tree constructed from an alignment of the 5S IGS sequence from the unknown sample (12-17-97b) with the top ten* sequence entries from a Blast search. Tree inference was constructed by using nearest neighbor interchange. Nodes indicate support after bootstrap replicates (500). Sequences from the top hits were from the following organisms: *Toxocara canis* (TCU65503), *Ascaris lumbricoides* (M27961.1), *Chandlerella quiscali* (HM641830), *Foleyella furcata (*AJ250988), *Mansonella ozzardi* (JF412308), *Onchocercidae* cf. *Mansonella ozzardi* (JF412312-13, JF412315-16). The closest match (indicated by the shortest horizontal distance of the branches in the tree) with respect to our sample, was sequence TCU 65503 from *Toxocara canis*, which was only 79.8% similar. Gaps and deletions in the alignment were treated as complete deletion and this only allowed for 262 nucleotide sites to be analysed. *(one sequence from *Litomosoides sigmodontis* LSU31639 was omitted as this was only 160 bp long and made the data in the analysis significantly less informative).

Blast search of the 285 bp sequence generated from the ITS PCR (JX014377) resulted in many close matches. Top hits were imported and aligned in Align X, prior to being exported to MEGA [12]. Neighbor-Joining analysis of these top matches clustered this sequence with Genbank entries AB110025 and AB571303, both molecules derived from *Toxocara cati*. The 285bp segment from the third PCR was 100% identical to these two entries, confirming the identity of this larval sample to be *Toxocara cati*.

We initiated a small survey of the six farms that had provided animals in the infected pooled batch. All six farms came from the same area in Ringsaker municipality in Hedmark county. A questionnaire; regarding production system, farm quality assurance scheme, feed practices and food storage, presence of cats on the farm as well as pest control; was sent to each of the six farms. The type of production varied between the farms: two were piglet producing herds; one had an integrated herd whilst the three remaining farms had fattening herds. All the farms were registered in the Norwegian farm quality assurance scheme (KSL) and reported that food was stored in closed silos and containers. Five of the six reported that cats were present on the farm, although only two of the farms actually owned cats. The cats were, on the whole, of unknown health status, with only one farm giving sporadic anthelmintic treatment to their older farm cat. Various rodent control measures were carried out on all six farms. Four used an externally sourced company whilst two put down poison themselves. Two of the farms, in addition to poison, reported trying to put up barriers around access points to prevent rodent access, and one used a cat for rodent control.

Further questions regarding the presence of cats and rodents in essential areas revealed that although cats were not allowed access to the production housing, two farms had seen cats in the food storage area or the straw bedding storage area during the six months prior to the survey. In addition half the farms reported seeing rodents in the pig housing during the course of the previous six months and half of the farms reported seeing rodents in the food storage area or the straw bedding during the previous six months.

This is the first report of *Toxocara cati* detected at meat inspection at a slaughterhouse. It was not possible to identify which of the animals had been infected and we were unable to narrow down the source of infection, but we know it originated from one of a total of six farms. The survey of these farms revealed that contamination of food or bedding material with cat faeces or infected rodents as well as the presence of infected rodents in pig housing, were all potential sources of infection. It is advisable to routinely investigate the parasitological status of any cats on farms and deworm, as necessary, to prevent the spread of parasitic infections. This finding also raises questions regarding the last reported cases of *Trichinella* in pigs in Norway. The presence of larvae in digests during *Trichinella* control does not necessarily confirm the detection of *Trichinella* larvae. Morphological confirmation, together with molecular species identification at the EU or National Reference Laboratory as required by EU legislation [1], is necessary to confirm a *Trichinella* diagnosis.

## Competing interests

The authors declare that they have no competing interests

## Authors' contribution

RKD carried out the morphological identification of the larva, designed and summarised the questionnaire, and helped prepare the manuscript. ØØ performed PCR, sequencing and molecular analysis of the larva and helped prepare the manuscript. AM carried out the digestion work at the meat inspection laboratory, and critically appraised the manuscript. All authors read and approved the final manuscript.
